# A SAW-Based Chemical Sensor for Detecting Sulfur-Containing Organophosphorus Compounds Using a Two-Step Self-Assembly and Molecular Imprinting Technology

**DOI:** 10.3390/s140508810

**Published:** 2014-05-19

**Authors:** Yong Pan, Liu Yang, Ning Mu, Shengyu Shao, Wen Wang, Xiao Xie, Shitang He

**Affiliations:** 1 State Key Laboratory of NBC Protection for Civilian, Research Institute of Chemical Defense, Yangfang, Changping District, Beijing 102205, China; E-Mails: Yangliujinjin@sina.com (L.Y.); Sdmuta@163.com (N.M.); Shaoshengyu@163.com (S.S.); 2 Institute of Acoustic, Chinese Academy of Science, Zhongguancun Street, Haidian District, Beijing 100080, China; E-Mails: Wangwenwq@mail.ioa.ac.cn (W.W.); Xiexiao08@mails.ucas.ac.cn (X.X.); Heshitang@mail.ioa.ac.cn (S.H.)

**Keywords:** surface acoustic wave (SAW), chemical sensor, self-assembled, molecularly imprinted (MIP), detection

## Abstract

This paper presents a new effective approach for the sensitive film deposition of surface acoustic wave (SAW) chemical sensors for detecting organophosphorus compounds such as O-ethyl-S-2-diisopropylaminoethyl methylphosphonothiolate (VX) containing sulfur at extremely low concentrations. To improve the adsorptive efficiency, a two-step technology is proposed for the sensitive film preparation on the SAW delay line utilizing gold electrodes. First, mono[6-deoxy-6-[(mercaptodecamethylene)thio]]-β-cyclodextrin is chosen as the sensitive material for VX detection, and a ∼2 nm-thick monolayer is formed on the SAW delay line by the binding of Au-S. This material is then analyzed by atomic force microscopy (AFM). Second, the VX molecule is used as the template for molecular imprinting. The template is then removed by washing the delay line with ethanol and distilled water, thereby producing the sensitive and selective material for VX detection. The performance of the developed SAW sensor is evaluated, and results show high sensitivity, low detection limit, and good linearity within the VX concentration of 0.15–5.8 mg/m^3^. The possible interactions between the film and VX are further discussed.

## 1. Introduction

**Figure 1.**
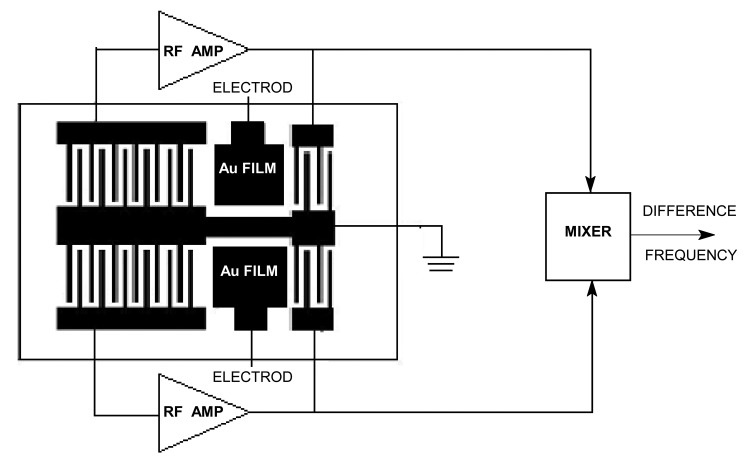
Schematic and principle of a SAW sensor.

Wohtjen [1] first reported the surface acoustic wave (SAW) method for gas sensing in 1979. Since then, SAW sensors have attracted great interest in the field of detection of poisonous and harmful gases at very low concentrations because of their small size, low cost, high sensitivity, and good reliability. The schematic and working principle of a SAW chemical sensor utilizing a dual-delay line oscillator structure is shown in Figure 1. SAW sensor responses arise from the mechanical interaction between the SAW and the sensitive film overlay. Thus, the adsorption efficiency of a sensitive film is a key factor affecting sensor performance, such as selectivity, sensitivity, stability, and response time. A SAW sensor responds to changes in mass on its surface with a shift in frequency and is thus most frequently used in gas-phase sensing applications. Accordingly, a chemically selective membrane should be used to collect and concentrate analyte molecules on the sensor surface by sorption. The sensitivity of a SAW sensor depends on the amount of vapor adsorbed and the inherent ability of the SAW transducer to respond to physical changes in the membrane caused by vapor adsorption. Therefore, we define sensitivity as the incremental change in signal occurring in response to an incremental change in analyte concentration, with detection limits depending on vapor sensitivity and on the noise of the sensor's signal.

Recognition layers are responsible for selective interactions with different types and forms of analytes. For instance, studies have shown polymers coated on SAW sensors are highly sensitive in qualitatively and quantitatively determining the composition of the gas [2], volatile organics [3], and chemical warfare agents [4–7]. For sensing purposes, SAW devices are coated with a molecular recognition or receptor layer, and adjoined to a frequency determining component. Depending upon the nature of a material, molecular recognition layers can be deposited onto the surface of SAW devices by spin coating, spray coating, sputtering, drop casting, electrospraying [8], or the Langmuir–Blodgett approach [9].

Self-assembled monolayer (SAM) technology provides an easy way of sensitive coating preparation with well-defined composition, structure, and thickness. Larry [10] reported, for the first time, a SAW chemical sensor for dimethyl methylphosphonate detection using SAW and self-assembled technology. Various substrates and functional groups have been investigated for SAM formation, including an alkanethiol monolayer on the Au surface. Although the growth mechanism of alkanethiol monolayers is unclear, alkanethiol SAMs are assumed to be formed as a result of chemical binding between the gold and sulfur atoms of thiol [11–13].

Molecular imprinting (MIP) technology has applications in syntheses, macromolecules, recognizability, and functional materials, among others [14]. The host-guest electronic, and steric complementarities, as well as host preorgnization, are three key elements in determining the stability of a complex in the gas phase [15]. By binding a monomer with a template molecule, a special three-dimensional structure could be formed after removing the template molecules in the sensitive coating. The target molecules could be successfully detected using this kind of selective and recognized coating [16,17]. Molecule host-guest systems, in which cyclodextrins (CDs) plays a very important role, are attracting increased interest. CDs are toroidal cyclic oligosaacharides with the secondary hydroxyls of glucose C-2 and C-3 on their more open face and the primary C-6 hydroxyl on the other face. The central cavities of CDs are hydrophobic or sterically restricted reaction fields; thus CDs are suitable host molecules. With a great variety of possible guests, this capacity has already been used in a number of applications [18,19]. β-CD is the most widely studied among the three CDs (α-, β-, γ-CD) because its cavity has the right size to bind a variety of aromatic and residues. In β-CD, the internal diameter of the cavity ranges from 6.0 to 6.5 Å, the average external diameter is about 15 Å, and the cavity depth is very close to 8 Å. As host-guest interactions can offer a novel approach to the functionalization of surface, thus, the chemisorbed layer of the host molecules offers a template upon which subsequent immobilization of guest molecules may occur. β-CD architecture, in which specific recognition reactions are used to build upon intricate structure in a controlled fashion, are possible by this method.

Although many technologies for sensitive film deposition have been successfully applied, most of them are inappropriate to prepare nanometer-thick films because of their poor film thickness, uniformity, stability, and reproducibility. Many advanced technologies such as LB require special equipment or are inadequate for certain polymers. In this paper, a simple way of preparing a self-assembled, molecularly imprinted coating for O-ethyl-S-2-diisopropylaminoethyl methylphosphonothiolate (VX) detection is proposed. First, mono[6-deoxy-6-[(mercaptodecamethylene)thio]]-β-CD is selected as the sensitive coating for VX detection and prepared on the gold surface of a SAW delay line by self-assembly. Second, VX molecule is used as a template for molecular imprinting. The developed SAW sensor using the new sensitive film deposition technique exhibits an excellent response to VX. The detection limit, selectivity, linearity and interference effect from the testing environment are experimentally evaluated.

## 2. Experimental Section

### 2.1. Reagents and Instruments

Mono[6-deoxy-6-[(mercaptodecamethylene)thio]]-β-CD was synthesized in our laboratory and analyzed by TOF-MS, FTIR, and NMR. O-Ethyl-S-2-diisopropylaminoethyl methylphosphonothiolate (Research Institute of Chemical Defence, Beijing, China). 1,10-decanedithiol (AR, TCI company, Tokyo, Japan), β-CD and mono(6-*o-p*-tolylsulfonyl)-β-CD (AR, Shanghai Chemical Reagent, Shanghai, China) and all other chemicals used were reagent grade.

As shown in Figure 1, dual SAW delay lines with separate frequencies were photolithographically fabricated on polished ST-quartz substrates. A 4 mm^2^ Au film for sensing layer coating was then prepared between the interdigital transducers. Using the prepared SAW delay lines as the feedback element, two delay line oscillators were developed, and the sensitive film was deposited onto one device for gas sensing, with the naked device acting as the reference. The mixed differential oscillation frequency was used to characterize the target species, and recorded by a Model Proteck C3100 Frequency Counter (Proteck Company, Incheon, Korea). The vapor cell for detecting VX is made of aluminium and SAW dual lines are inserted in it, the cell is about 30 × 25 × 6 mm, there is an air hole on both sides of the cell so that VX could pass through by pumping, and the velocity of flow is 0.6 L/min.

### 2.2. Synthesis of Mono[6-deoxy-6-[(mercaptodecamethylene)thio]]-β-CD

**Scheme 1.**
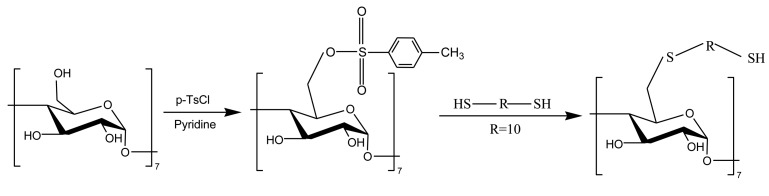
Synthesis of mono[6-deoxy-6-[(mercaptodecamethylene)thio]]-β-CD.

The prepared β-CD was dried for 24 h at 100 °C in vacuum prior to utilization. Pyridine and N,N-dimethylformamide were distilled from CaH_2_. Mono(6-*o-p*-tolylsulfonyl)-β-CD was prepared according to [20,21], and the crude product was recrystallized twice from acetone. Then, the product was reacted with 1,10-decanedithiol in an aqueous solution of Na_2_CO_3_ containing 20% ethanol at 50 °C to yield mono[6-deoxy-6-[(mercaptodecamethylene)thio]]-β-CD. The crude product was purified on a reversed-phase column. The synthesis route is depicted in Scheme 1.

### 2.3. Preparation of a Self-Assembled, Molecularly Imprinted Film of SAW Sensor

**Figure 2.**
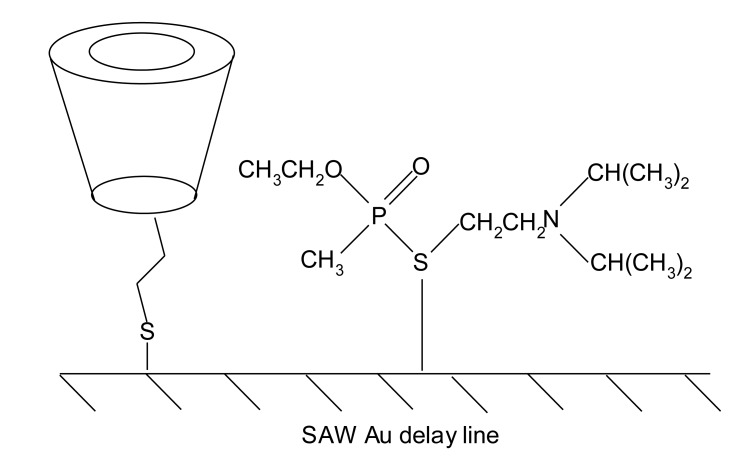
Competition between self-assembled film and molecular template on the surface of SAW Au delay line.

Mono[6-deoxy-6-[(mercaptodecamethylene)thio]]-β-CD is a kind of alkanethiol, and VX is also a dialkyl sulfide. Both react very well on the Au surface to form Au-S bonds, so the Au surface is not entirely occupied by mono[6-deoxy-6-[(mercaptodecamethylene)thio]]-β-CD (Figure 2). Obviously, the self-assembled, molecularly imprinted film was not perfectly prepared.

To solve this problem, we prepared a self-assembled, molecularly imprinted sensitive film by a two-step technology. The first step was the self-assembly of mono[6-deoxy-6-[(mercaptodecamethylene)thio]]-β-CD on the Au surface. The second step was molecular imprinting between the VX template and mono[6-deoxy-6-[(mercaptodecamethylene)thio]]-β-CD.

**Figure 3.**
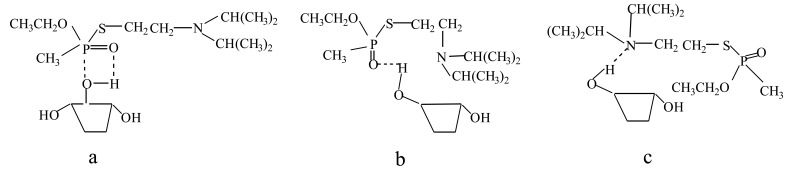
Reaction between self-assembled imprinted film and VX.

Prior to depositing the sensitive film, the Au surface of SAW delay lines were purged with a 3:1 (V/V), piranha (sulfuric acid, hydrogen peroxide) solution for cleaning before immersing into a 1 × 10^−4^ M mono[6-deoxy-6-[(mercaptodecamethylene)thio]]-β-CD ethanol solution for 24 h. Then, the SAW delay lines were washed with ethanol, and immersed into a 10 mM VX ethanol solution for another 24 h, VX may react with mono[6-deoxy-6-[(mercaptodecamethylene)thio]]-β-CD in different binding ways, as shown in Figure 3a–c. After accomplishment of the binding between the template VX and mono[6-deoxy-6-[(mercaptodecamethylene)thio]]-β-CD, the SAW delay lines were washed with ethanol and distilled water to remove the VX templates. Consequently, the self-assembled, molecularly imprinted film was successfully formed on the Au surface of the SAW delay line.

## 3. Results and Discussion

### 3.1. Analysis by AFM and Calculations

**Figure 4.**
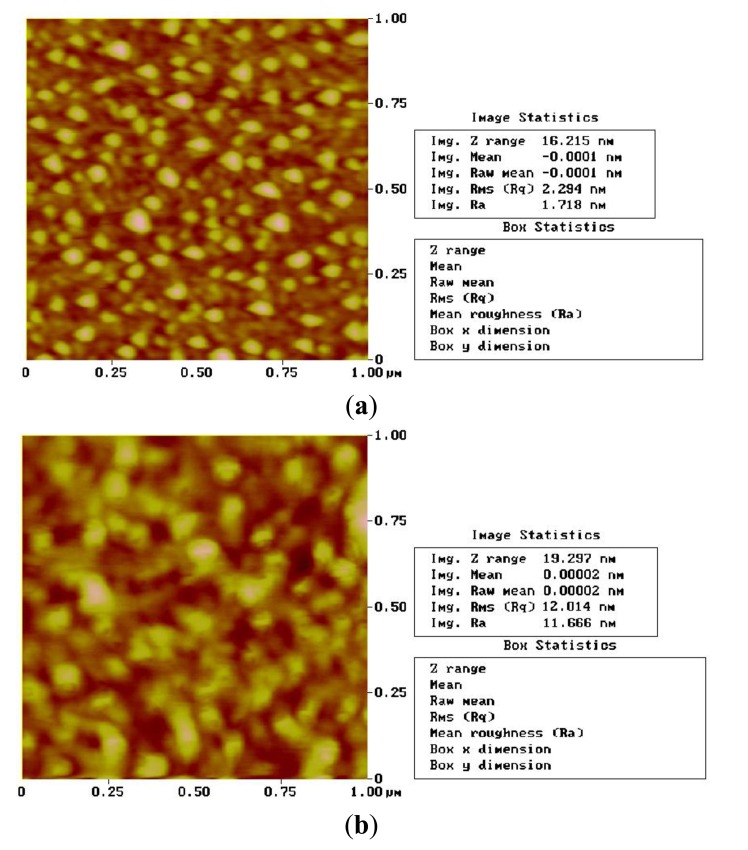
The 3D surface AFM photograph of SAW Au delay line. (**a**) Bare Au delay line, RMS [Rq] = 2.294 nm; (**b**) After self-assembled procedure, RMS [Rq] = 12.014 nm.

AFM analysis was used to evaluate the covalent bonding and appearance of the self-assembled film surface, as shown in Figure 4. The RMS [Rq] of Au with no mono[6-deoxy-6-[(mercaptodecamethylene)thio]]-β-CD was only 2.294 nm, and this value increased to 12.014 nm when the Au delay line was coated. Obviously, mono[6-deoxy-6-[ (mercaptodecamethylene)thio]]-β-CD was successfully formed on the Au surface of the SAW device.

Given the high mass-sensitivity of the SAW sensor, the thickness of the self-assembled, molecularly imprinted film was estimated by measuring the frequency shift as follows [22,23]:

(1) $$\Delta \text{f}=-1.26\times 10^{6}{{\text{f}}_{0}}^{2}\text{h}\rho$$

where Δf (Hz) is the frequency shift between coated and uncoated SAW delay lines. f_0_ (MHz) is the operating frequency of the SAW sensor. h (cm) is the film thickness of the self-assembled, molecularly imprinted film, and ρ (g/cm^3^) is the density of the film material.

The frequency of the SAW sensor was set to 300 MHz, the density of mono[6-deoxy-6-[(mercaptodecamethylene)thio]]-β-CD was 1.5 g/cm^3^, and Δf was measured to be 10 kHz, so the thickness of SAM was estimated to be 2 nm on average.

### 3.2. Analysis of the MIP Effect

**Figure 5.**
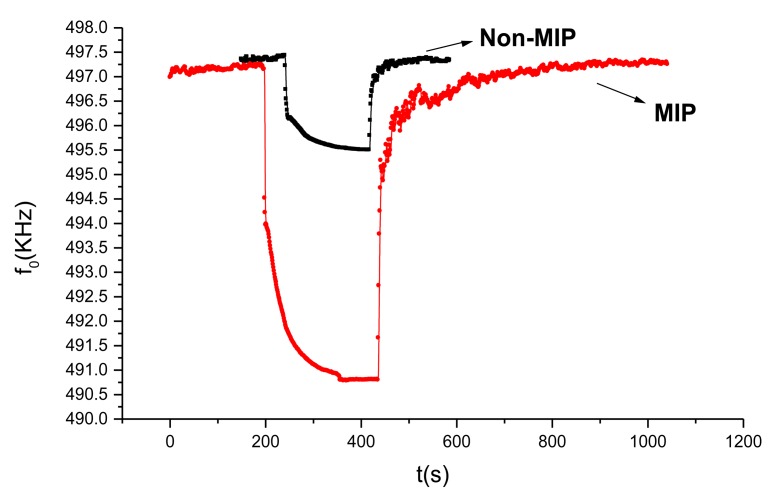
Confirmation of MIP effect (28 °C, RH = 70%).

The MIP effect was investigated by comparing the response to VX of the MIP-coated sensor with that of the non-MIP-coated sensor, and the results are shown in Figure 5. When the developed non-MIP coated SAW sensor was exposed to 1.5 mg/m^3^ VX, a very weak frequency response of only 2 kHz was observed because of the lack of particular 3D space structures. After MIP, as the molecular imprinting approached the covalent process, the covalent imprinting mainly depended on an easily cleavable arrangement between the template and the monomeric compound, which induced cavity formation. Given the imprinting effect, when VX was detected using the MIP-coated sensor, about 7 kHz frequency shift was detected. Moreover, a larger frequency response was obtained over the non-MIP-coated SAW sensor, hence, hence, the MIP effect was experimentally confirmed.

### 3.3. Sensor Response to VX Detection

Table 1 shows that VX was detected under different test conditions. With decreased VX concentration, the sensor frequency decreased and a longer response time was observed. At high concentrations, much more VX molecules in the unit gas phase were adsorbed by the mono[6-deoxy-6-[(mercaptodecamethylene)thio]]-β-CD coating, so the time to reach equilibrium was shortened. On the contrary, at low concentrations, lower gas molecule adsorption led to a relatively low sensor response. Establishment of the response equilibration also took a long time, which resulted in a long response time. According to IUPAC methods, the detection limit toward VX was evaluated as 0.15 mg/m^3^ (S/N > 3), and the linearity response to VX ranged within 0.15–5.8 mg/m^3^.

**Figure 6.**
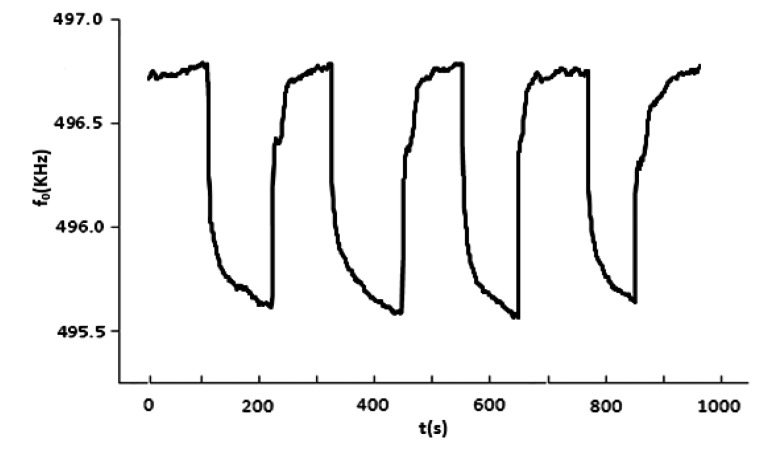
Response curve for four successive exposures of the sensor to VX at 1.25 mg/m^3^.

**Figure 7.**
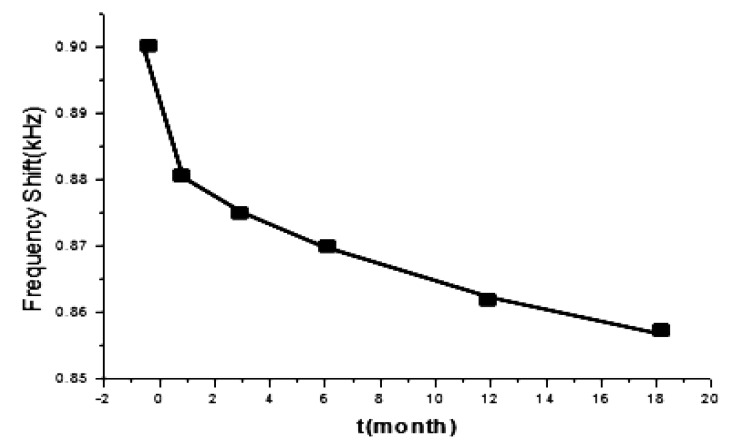
Stability study of MIP sensor.

To study the stability of this sensor system, the MIP sensor was kept at room temperature, the typical response curve for four successive VX exposures is shown in Figure 6. And VX was detected with it under the same condition for many months (Figure 7). In the first 6 months, the detection signal for VX decreased by about 3.3%, after 18 months, the decrease reached about 4.4% and then abated, so VX could still be detected with the MIP sensor.

### 3.4. Anti-Interference Experiment of the SAW-MIP Sensor

To further confirm the MIP effect, many other gases with larger concentrations (100–1000 times that of VX) were used to perform an anti-interference experiment. The results are listed in Table 2. Almost no influence on the sensor response was observed from most common organic solvents and gases. However, organic amines and organic acids at high concentrations obviously affected the sensor because they exhibited extend binding or adsorptivity on the surface of sensitive film. Nevertheless, the resulting frequency shifts were much lower than that of VX.

## 4. Conclusions

The physicochemical properties of a chemoselective material are very critical to performance improvement in chemical sensing applications. In particular, the chemical preparation technique for sensitive films affects the coating uniformity, adhesion, and quality of the sensor. So many chemoselective coatings for functionalized self-assembled monolayer structures have been utilized to develop SAW chemical sensors that can exhibit a very rapid response at extremely low concentrations.

In this work, a novel two-step self-assembly and molecular imprinting technology for preparing sensitive films used to detect warfare agents VX was developed. The technology produced a film containing covalently immobilized onto host molecules (mono[6-deoxy-6-[(mercaptodecamethylene)thio]]-β-CD). The SAW sensor coated with the self-assembled, molecularly imprinted film was very sensitive to VX, and a significant frequency response was observed. High sensitivity and excellent selectivity were obtained because of the host-guest interaction between mono[6-deoxy-6-[(mercaptodecamethylene)thio]]-β-CD cavity and VX.

Overall, our results indicated that mono[6-deoxy-6-[(mercaptodecamethylene)thio]]-β-CD with good chemical selectivity can be successfully designed using appropriate molecules for the formation of self-assembled, molecularly imprinted films on SAW gold delay line. Related works are currently in progress in our laboratory.

## Figures and Tables

**Table 1.** Response of different concentrations to VX (20 °C, RH = 50%).

**Concentration (mg/m^3^)**	**Frequency Shifts (Hz)**	**Response Time (min)**	**Recovery Time (min)**
5.80	2850	1.7	4.5
4.30	2460	1.9	4.3
2.67	1715	2.3	4.1
1.60	1507	2.4	3.5
0.85	1120	2.8	2.9
0.55	860	5.6	2.3
0.15	437	8.5	1.1

**Table 2.** Response of the SAW-MIP sensor to interferences.

**Interference Gas**	**Concentration (mg/m^3^)**	**Frequency Shifts (Hz)**
Omethoate	1,000	1,123
CH_3_OH	10,000	168
CH_3_CH_2_OH	10,000	123
HCOOH	1,000	420
CH_3_COOH	1,000	360
CH_3_(CH_2_)_4_COOH	1,000	1,360
NH_3_	2,000	403
C_6_H_5_NH_2_	2,000	516
O-Anisidine	1,000	212
C_2_H_5_OC_2_H_5_	10,000	103
Petrdeumether	10,000	169
THF	10,000	230
n-C_6_H_14_	10,000	197
n-C_8_H_18_	1,000	341
CCl_4_	10,000	214
HCHO	1,000	125
CH_3_COCH_3_	10,000	118
CH_3_COOC_2_H_5_	10,000	103
C_6_H_6_	10,000	189
C_6_H_5_CH_3_	1,000	226
C_6_H_5_Cl	1,000	103
H_2_O	10,000	2,213
CH_3_CN	1,000	205
Smog	high	-
